# Primary non-adherence to inhaled medications measured with e-prescription data from Poland

**DOI:** 10.1186/s13601-020-00346-7

**Published:** 2020-10-07

**Authors:** Grzegorz Kardas, Michał Panek, Piotr Kuna, Przemysław Kardas

**Affiliations:** 1 Department of Internal Medicine, Asthma and Allergy, Medical University of Lodz, Lodz, Poland; 2 First Department of Family Medicine, Medical University of Lodz, Lodz, Poland

**Keywords:** Inhaled medications, Inhalators, Asthma adherence, COPD adherence, Primary non-adherence, e-prescription

## Abstract

**Background:**

Treatment adherence greatly influences the clinical outcomes in various fields of medicine, including management of asthma and COPD. With the recent implementation of a nationwide e-Health solutions in Poland, new and unique opportunities for studying primary non-adherence in asthma and COPD emerged. The aim was to study primary non-adherence to inhaled medications available in Poland indicated in asthma and/or COPD and analyse the impact of patients’ demographics and inhalers’ characteristics (dry powder inhalers (DPIs) vs metered dose inhalers (MDIs) and presence of a dosage counter) on primary non-adherence.

**Methods:**

A retrospective analysis of all e-prescriptions issued in Poland in 2018 (n = 119,880) from the national e-prescription pilot framework.

**Results:**

Primary non-adherence for inhalable medications reached 15.3%. It significantly differed among age groups—the lowest (10.8%) was in 75 + years-old patients, highest (18%) in 65–74 years-old patients. No gender differences in primary non-adherence were found. The highest non-adherence was observed for ICS + LABA combinations (18.86%). A significant difference was found between MDI and DPI inhalers and between inhalers with/without a dosage counter.

**Conclusions:**

Out of e-prescriptions for inhaled medications issued in 2018 in Poland, 15.3% were not redeemed. The degree of primary non-adherence was influenced by age, but not gender. Significant differences between MDIs and DPIs and between inhalers with/without a dosage counter were observed.

## Introduction

Treatment adherence greatly influences the clinical outcomes in various fields of medicine. In general, the worse is the adherence, the worse are the health outcomes and patients’ quality of life [1]. It is also a major determinant of healthcare costs [2]. These effects also take place in the management of obstructive lung diseases—asthma and chronic obstructive pulmonary disease (COPD). In course of these two conditions, the primary clinical role is attributed to inhaled medications, which are important in management of disease symptoms and natural course of the diseases.

As asthma and COPD are frequent and most often chronic conditions, the key to their successful management comes with systematic drug use, particularly those inhaled. Specific drug selection depends on the diagnosis and indication, previous treatment response, disease severity, patient’s individual needs and preferences and various other patient-related factors. The inhaled drugs used in these two indications include: inhaled corticosteroids (ICS), short-acting beta-agonists (SABA), long-acting beta-agonists (LABA) and long-acting muscarinic antagonists (LAMA) [3].

Asthma and COPD symptoms—their type and severity—often vary among patients and also may change over time. Although these diseases are life-threatening conditions—especially during an exacerbation or severe breathlessness attack—in course of chronic treatment patients still have some degree of freedom in self-management during stable, controlled periods of their disase. Despite that, efficient long-term asthma and COPD treatment comes from the proper and regular use of inhaled medications. In this case, adherence to treatment, and primary adherence in particular, greatly contributes to the therapeutic success.

Defined in the ABC European consensus, medication adherence is as an active, cooperative and voluntary participation of the patient in following recommendations from a healthcare provider. The process involves three crucial steps:

- Initiation—defined as the moment the first dose is taken by the patient;
- Implementation—defined by the extent of prescription regimen fulfilment;
- Discontinuation—that is when the patient discontinues taking the prescribed drugs [4].

In general, primary non-adherence is a situation when a patient does not obtain the prescribed drug from the pharmacy during the prescription’s validity [5]. Having assumed that a prescription for an individual drug is a proof that the need for pharmacotherapy has been confirmed by a medical professional, primary non-adherence is a major discordance from the treatment schedule. Many studies have covered aspects of primary non-adherence, particularly in terms of chronic diseases’ management (e.g. hypertension, diabetes mellitus and other) [6–9]. However, data on primary non-adherence in treatment of asthma and COPD is limited. Moreover, the issue of general primary non-adherence and of that for inhaled medications, has yet not been properly studied in Poland because reliable data was lacking. Fortunately, the recent implementation of nationwide e-Health solutions in Poland created new and unique opportunities for studying primary non-adherence in asthma and COPD.

## Methods

The aim was to study the primary non-adherence to all of the inhaled medications available in Poland (as of December 2018) with indication for asthma and/or COPD—overall and for individual drugs. Additionally, the impact of patients’ demographics on this phenomenon was analysed. The variation of primary non-adherence across different types of inhalers—(dry powder inhalers (DPIs) vs metered dose inhalers (MDIs)) and presence of a dosage counter within the inhaler was also studied.

We retrospectively analysed the data from all of the e-prescriptions issued in Poland in 2018 (n = 119,880) from the national e-prescription pilot. The data came from 43 medical units (primary care, hospitals and specialist clinics from 9 out of 16 voivoidships of Poland). The healthcare centres participating in this pilot programme were invited by CSIOZ (see below) or voluntary joined voluntarily. The e-prescriptions were prescribed by 190 doctors of various specialisations.

The study database was provided to the researchers by the Center of Information Systems for Healthcare (Centrum Systemów Informacyjnych Ochrony Zdrowia, CSIOZ)—a Polish governmental institution working on the Polish healthcare system digitalization.

A recent task of this institution was the implementation of the nationwide system of e-prescriptions, which is fully operational as of January 2020.

The data used in the analysis was fully anonymized. Thus, the study was not subject to ethical approval, according to the Ethical Commission of Medical University of Lodz. The records included basic patient characteristics (age and gender), the date of prescription issuing, prescription details (drug trade name, dosage, packages number), and details and date of drug dispensation (only if it happened). In the literature, primary non-adherence is generally defined as not obtaining the medication within the defined number of days after prescribing [5]. However, the database used in this study did not include exact clinical data (that is individual patient’s diagnosis) and the long-term, individual prescription histories were not possible to be studied. Therefore, for this study purpose, we defined primary non-adherence as not obtaining an individual e-prescription from the pharmacy within 30 days, as it is the general prescription validity in Poland—including inhalable medications prescriptions.

In our analysis, we included all of the inhaled medications available in Poland as of December 2018, with all of their available doses and formulations (21 compound combinations, over 100 available preparations - the full list of analysed ATC codes is available in Table 1). Each of the available inhaled medications was categorized by the authors according to their characteristics as either DPI, MDI, metered dose liquid inhaler (MDLI) or nebulization.

**Table 1:** List of the compounds analyzed (with ATC codes)

No.	Drug class	ATC code and drug name
1.	ICS	R03BA01 Beclometasone
2.	ICS	R03BA02 Budesonide
3.	ICS	R03BA05 Fluticasone
4.	ICS	R03BA08 cyclesonide
5.	ICS + LABA	R03AK06 fluticasone + salmeterol
6.	ICS + LABA	R03AK07 Budesonide + formoterol
7.	ICS + LABA	R03AK08 Beclometasone + formoterol
8.	LABA	R03AC13 formoterol
9.	LABA	R03AC12 salmeterol
10.	LABA	R03AC18 indacaterol
11.	LAMA	R03AL06 clicopironium
12.	LAMA	R03BB01 Ipratropium
13.	LAMA	R03BB04 Tiotropium
14.	LAMA	R03BB07 Umeclidynium
15.	LAMA + LABA	R03AL03 Ipratropium + salbutamol
16.	LAMA + LABA	R03AL04 Indacaterol + glycopyrronium
17.	LAMA + LABA	R03AL04 Olodoterol + tiotropium
18.	LAMA +LABA	R03AL03 Umeklidynium + wilanaterol
19.	SABA	R03AC02 Salbutamol
20.	SABA	R03AC04 Fenoterol
21.	SABA + LAMA	R03AK03 Fenoterol + ipratropium

First, the descriptive statistics of overall prevalence of primary non-adherence were calculated. Following that, the potential drivers of primary non-adherence (age and gender) were studied. Age was categorized into 5 groups: 1–18, 19–39, 40–64 years, 65–74 and 75 + years. Categorical variables were expressed as proportions and compared between the groups using the χ^2^ test. The statistics were calculated using the Statistica 10 software (TIBCO Software Inc., USA). A *p* value of < 0.05 was considered significant.

## Results

Out of all (119,880) individual drugs prescribed on e-prescriptions in Poland in 2018, 1973 (1.6%) were inhalable medicines of interest for this study. The primary non-adherence for inhalable medicines reached 15.3%, as 1671 (84.7%) of e-prescriptions on those drugs were obtained by the patients.

995 (50.4%) e-prescriptions for inhalable drugs were prescribed for males. Primary non-adherence among males reached 16.7% and it was not significantly different from that among females (13.9%, p = 0,086) (Table 2). Moreover, none of the analysed inhaled medications was significantly more often redeemed by either of genders.

**Table 2:** Levels of primary non-adherence to inhaled medicines by gender, chi^2^ = 2935; p = 0,086

Patient	Gender				Summary	
	Male		Female			
	N	%	N	%	N	%
Adherent	829	83.3	842	86.1	1671	84.7
Non-adherent	166	16.7	136	13.9	302	15.3
Summary	995		978		1973	

**Fig. 1:**
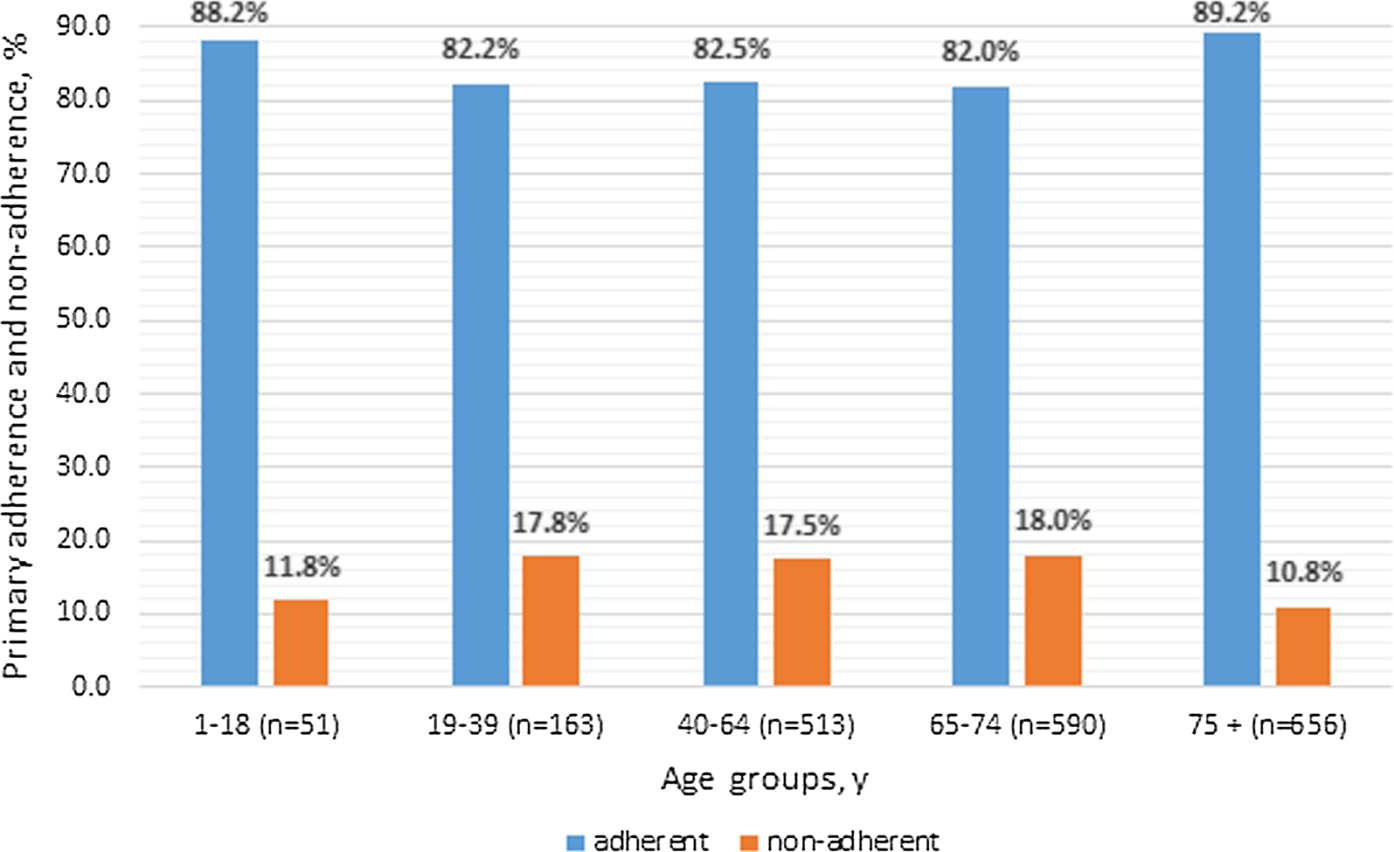
Levels of inhaled medications primary adherence/non-adherence among age groups. Chi^2^ = 16,641; p = 0,0023

The patients who obtained their inhalable medicine e-prescriptions were slightly, but significantly older on average than those who did not (65.8 ± 18.0 vs. 64.1 ± 17.1 years, respectively, p < 0.05). The further analysis of age-related primary non-adherence dependencies has shown significant differences between age groups. The highest primary non-adherence (18.0%) was observed among patients aged 65-74, whilst the highest primary adherence (89.2%) was among the 75 + years-old patients (Fig. 1).

The extents of primary non-adherence to individual inhaled medication drug classes is shown in Table 3. The most commonly prescribed groups were LABAs (422 e-prescriptions, 16.82% non-adherent) and ICSs (419, 13.37% non-adherent).

**Table 3:** Primary non-adherence for inhaled medication drug classes

Drug class	Issued	Redeemed	Primary adherence [%]	Primary non-adherence [%]
ICS	419	363	86.63	13.37
ICS + LABA	350	284	81.14	18.86
LABA	422	351	83.18	16.82
LAMA	352	303	86.08	13.92
LAMA + LABA	41	37	90.24	9.76
LAMA + SABA	1	1	100	0
SABA	321	274	85.36	14.64
SABA + LAMA	67	58	86.57	13.43

For drugs of interest in this study, the mostly prescribed (346) was a LABA—formoterol. Primary non-adherence to this drug was 19.08%. Among SABAs, the most frequently prescribed was salbutamol—274 e-prescriptions, 14.6% primary non-adherence. The most frequently prescribed LAMA was tiotropium—115 e-prescriptions, 16.52% primary non-adherence. The values for all of the analysed inhalable drugs are presented in Table 4.

**Table 4:** Primary non-adherence to individual inhaled medications

Drug Class	ATC code and drug name	Issued	Redeemed	Primary adherence [%]	Primary non-adherence [%]
ICS	R03BA01 Beclometasone	–	–	–	–
ICS	R03BA02 Budesonide	268	230	85,82	14.18
ICS	R03BA05 Fluticasone	48	41	85,42	14.58
ICS	R03BA08 Cyclesonide	103	92	89,32	10.68
ICS + LABA	R03AK06 Fluticasone + salmeterol	178	157	88,20	11.8
ICS + LABA	R03AK07 Budesonide + formoterol	88	58	65,91	34.09
ICS + LABA	R03AK08 Beclometasone + formoterol	84	69	82,14	17.86
LABA	R03AC13 Formoterol	346	280	80,92	19.08
LABA	R03AC12 Salmeterol	72	67	93,06	6.94
LABA	R03AC18 Indacaterol	4	4	100,0	0
LAMA	R03AL06 Glicopironium	19	16	84,21	15.79
LAMA	R03BB01 Ipratropium	209	183	87,56	12.44
LAMA	R03BB04 Tiotropium	115	96	83,48	16.52
LAMA	R03BB07 Umeclidynium	9	8	88,89	11.11
LAMA + LABA	R03AL03 Ipratropium + salbutamol	1	1	100,0	0
LAMA + LABA	R03AL04 Indacaterol + glycopyrronium	27	25	92,59	7.41
LAMA + LABA	R03AL04 Olodoterol + tiotropium	6	5	83,33	16.67
LAMA + LABA	R03AL03 Umeklidynium + wilanaterol	8	7	87,50	12.5
SABA	R03AC02 Salbutamol	274	234	85,40	14.6
SABA	R03AC04 Fenoterol	47	40	85,11	14.89
SABA + LAMA	R03AK03 Fenoterol + Ipratropium	67	58	86,57	13.43

Further analysis was focused on differences in primary non-adherence in relation to inhaler type (DPI vs MDI) and characteristics (presence of dosage counter). A statistically significant difference was found between DPIs (995 e-prescriptions) and MDIs (759 e-prescriptions), for which the extents of primary non-adherence were 17.0% and 13.4% respectively. Within drug class groups, where comparison was possible (ICSs, ICS + LABAs, LABAs and LAMAs), that is for which both types of inhalers within a group were available, there were no statistically significant differences in primary non-adherence. Among MDIs and DPIs, the inhalers without a dosage counter had significantly lower primary non-adherence than those with it - 13.4% vs. 17.0% respectively (chi^2^ = 4145; p = 0,0415). Within MDI and DPI groups such differences were not significant.

## Discussion

Inhalable medicines are the established basis of asthma and COPD management according to GINA and GOLD recommendations [3, 10]. Depending on the diseases’ course and severity, a proper treatment scheme of asthma/COPD should be implemented, yet still a common part of these treatments are always inhaled drugs. Those drugs are effective in reducing the respiratory symptoms and in long-term disease management. Moreover, their effects are crucial in exacerbation prevention and reduction of life-threatening incidents and mortality rate. Nonetheless, in proper management of asthma and COPD patient adherence is a greatly contributing factor [11, 12]. Medication adherence is considered a major factor contributing to asthma/COPD exacerbations, mortality and disease course [13, 14].

According to the literature, adherence is modified by numerous factors: socio-economic (e.g. family and employment), healthcare system-related (e.g. drug information and administration), condition-related (e.g. symptoms or lack of them), drug-related (e.g. drug regimen, formulation and costs) and importantly–patient-related (e.g. level of education, mental and psychological condition, health beliefs and concerns, cognitive functions) [15–17]. In particular, studies show that adherence in COPD is device-related, with the device design resulting in under- or overuse, depending on its technical characteristics (dosage counter, the possibility to load an inhalation dose without real inhalation) [18]. Moreover, studies show that in asthma adherence is dependent on patient treatment beliefs and perception [19].

In order to assess the patients’ adherence, a number of methods may be implemented. These include direct (e.g. drug or biomarker blood concentration) or indirect methods (e.g. pill counts, database research, self-reports) [20]. The use of e-prescription databases is subject to minimized bias in assessment of primary non-adherence, since the prescription drugs may legally only be obtained when a patient possesses a prescription and fills it at a pharmacy. Self-report measures are considered not sufficiently precise and unreliable compared to other methods. Pill or dosage counts possibly overestimate the exact doses taken, as patients may influence the amount of those left in the package [21–23]. However, to properly assess the primary adherence in community setting, no other method than database search provides the most accurate data [24].

Depending on the definition, setting and methodology used, primary non-adherence to various drugs reaches different levels. In accordance to definition used in this study, it reaches a wide range of extents in different settings. In an analysis performed in the USA by Rutherford et al. four important drug groups—antihypertensives, lipid-lowering agents, hypoglycemics, and antidepressants—were found to reach a mean level of 14.6% primary non-adherence [25], whereas e.g. for dermatological drugs primary non-adherence reached 24.7% [26].

Few studies have covered the issue of inhaled medications primary non-adherence. Yet some examples addressing this phenomenon may be found in the literature. With an approach similar to ours, Fischer et al. analysed primary non-adherence using data from 195,930 e-prescriptions from the United States. For “asthma medications”, the primary non-adherence level reached 19.9% in adults aged 19 + and 11.4% in children. For newly prescribed drugs in this field it reached 25.1% in adults and 11.3% in children. Similarly to our approach, the authors of that study analysed only the population of patients that used the e-prescriptions. In another study, also by Fischer et al., primary non-adherence to “Antiasthmatic and bronchodilator agents” medications reached 17.9% [27, 28].

A meta-analysis of 31 articles on primary non-adherence published in 2019 by Cheen et al. summarized the results of 6 studies in asthma/COPD area published in years 2009–2014. The levels of primary non-adherence to the asthma/COPD medications ranged between 9 to 25%, with an average of 14.0%. In comparison, for other therapeutic areas covered in this meta-analysis, the primary non-adherence reached 25.0% for osteoporosis, 16.0% for hypertension, 10.0% for diabetes, 25.0% for hyperlipidemia and 12.0% for depression (17.0% across all groups). The authors also indicated several factors significantly associated with primary non-adherence, in particular in asthma/COPD, of which positively correlated older age and male gender and higher co-payment. Interestingly, the authors did not confirm a dependence resulting from differences in dosage forms [29].

The impact of inhaler type (MDI vs DPI) on primary non-adherence has been subject of only few analyses up to date. In 2014 van Boven et al. used a Dutch pharmacy dispensing data from 1994 to 2012 in order to analyse LABA persistence in COPD patients. The authors of that study found no significant differences between MDIs and DPIs [30]. We believe the differences observed in our study might be a result of generally lower out-of-pocket costs of MDIs, as compared to DPIs. Of a note is that in Polish healthcare system, patients pay various drugs co-payments (with varying drug reimbursement levels: 100%, 70%, 50%, 0% or a standard co-payment of 3.20 PLN per package), that are dependent on the drug, indication, patient’s age and other.

A number of interesting results on primary non-adherence to inhaled medications have been presented in this paper. Using the data of highest possible quality available to date, that originated from a nationwide e-prescription database, a specific level of primary non-adherence to inhaled medications in Poland was proven. The non-adherence to these drugs was lower than obtained in our previous study on drivers of general non-adherence in Poland, where for drugs in 6 major areas (antidiabetic, antithrombotic, cardiovascular, cholesterol medications, antibiotics and psychiatric drugs) the primary non-adherence was 20.8% [31]. Also, it was lower than of that for antihistamine drugs, for which the level of primary non-adherence was 21% [32].

Importantly, this is the very first study that covered primary non-adherence to inhaled medications in Poland and also one of the very few such studies worldwide. A certain limitation of this study came from the database structure, as it was not possible to study the exact clinical reasons of each e-prescription. The data was anonymous and no additional clinical data (in particular, the patient’s diagnosis) were available. However, this study can still be considered an objective measure of adherence in obstructive diseases, since the analysed drugs’ approved indications (as reflected in their Summaries of Product Characteristics) include only asthma and COPD management. Also noteworthy is the fact that during data collection, the new e-healthcare system in Poland was a pilot solution, and thus the primary non-adherence results may have been influenced. Despite that, we believe the data used in this study is still of the highest possible quality and minimally biased. It was not self-reported nor dependent on any physicians’ opinion on patients’ non-adherence. As the lowest degree of primary non-adherence concerned patients aged 75 + , the common perception of a possible technological barrier of an e-prescription system for the eldest cannot be proven. The study database originated from a nationwide pilot e-prescription programme, thus it can be considered complete.

A further study limitation was that it was only possible to analyse the primary non-adherence, that is studying the act of obtaining/not obtaining a particular e-prescription. The number of doses a patient took or skipped was also not measured. This issue could not be analysed with the data used in this study and, in fact, this was not an objective of this study. A longitudinal analysis of a particular patient was also not possible to be performed with the analysed dataset.

Finally, we could not analyse the exact reasons behind the primary non-adherence, which could have been diverse: disbelief in diagnosis or physician, drug characteristics and other [15]. We also could not analyse the impact of patients’ out-of–pocket costs (in Poland these are dependent on indication, age and having a national health insurance) on primary non-adherence, since the database did not include the data on that subject.

The use of e-prescription is rising recently, both in Europe and worldwide. Studies on e-prescription systems in Europe show their multi area benefits: health, economic, social, patient-oriented and other. Major health benefits include reduced medication errors, better medicine accessibility and, what we recognize as crucial in therapy—increased monitoring of adherence. The economic benefits include efficiency gains for healthcare professionals, better transparency, reduced frauds and printing costs. The social profits concentrate around patient satisfaction, financial relief and assistance for the elderly [33]. Patients using e-prescriptions gain a possibility to trace their medication history better via a patient on-line portal. Finally, e-prescriptions help patients to adapt to other tele-health solutions, such as teleconsultations, and are of great help in case of limited physical access to healthcare facilities (e.g. recent coronavirus outbreak).

Regardless of why patients are non-adherent to inhaled medications or other drugs, some corrective solutions are described. In a randomized trial of allergic rhinitis treatment with intranasal corticosteroid treatment, a daily short message service reminder improved patient adherence [34]. Another SMS service for asthma patients that reminded about their daily inhaled medications was effective and increased adherence by 17.8% [35]. Such an approach–an SMS reminder to obtain an e-prescription would possibly better primary adherence. As the e-prescription solution in Poland in fact includes SMS service, this approach could be simply implemented with a reminder of a particular e-prescription expiration date approaching.

A study of improving adherence to ICSs in asthma by Vollmer et al. has shown a small, yet significant improvement with an interactive voice recognition phone calls system that reminded patients of their medication refills and continuous ICS treatment. Such system, if fact similar to SMS service, could also improve primary adherence in pair with e-prescription system [36]. Other approaches described in the literature include mobile apps that stress the significance of proper clinical allergy diagnosis and further encourage patients’ adherence [37–39].

In order to better picture the observed phenomenon, in our future research we intend to further broaden the analysis spectrum. This will be achieved by inclusion of higher number e-prescription databases, obtained in 2019 and further. Since from January 2020 the e-prescription is the applicable standard of drug prescribing in Poland, in near future we hope to provide even more objective and fully-nationwide results.

## Conclusions

In our study more than 1 out of 7 e-prescriptions to inhaled medications were not obtained by the Polish patients. The degree of primary non-adherence to these drugs was influenced by age and not by gender. The highest non-adherence was observed for ICS + LABA combinations (18.86%). Particular compounds had different primary non-adherence levels reaching 34.09% for budesonide + formoterol combination. Significant differences in primary non-adherence between MDI and DPI inhalers and between inhalers with/without a dosage counter were found. To authors’ knowledge, this study is the first to cover primary non-adherence to inhaled medications in Poland and one of the very few such studies worldwide.

## Data Availability

The data set is available from the corresponding author upon reasonable request.

## Abbreviations

COPD: Chronic obstructive pulmonary disease
DPI: Dry powder inhaler
ICS: Inhaled corticosteroid
LABA: Long-acting beta-agonist
LAMA: Long-acting muscarinic antagonist
MDI: Metered dose inhaler
MDLI: Metered dose liquid inhaler
SABA: Short-acting beta-agonist
